# Predictions of the Length of Lumbar Puncture Needles

**DOI:** 10.1155/2014/732694

**Published:** 2014-08-13

**Authors:** Hon-Ping Ma, Yun-Fei Hung, Shin-Han Tsai, Ju-chi Ou

**Affiliations:** ^1^Emergency Department, Shuang Ho Hospital, Taipei Medical University, No. 291, Zhongzheng Road, Zhonghe District, New Taipei City 235, Taiwan; ^2^Department of Emergency Medicine, School of Medicine, College of Medicine, Taipei Medical University, Taipei, Taiwan; ^3^Graduate Institute of Injury Prevention and Control, College of Public Health and Nutrition, Taipei Medical University, Taipei, Taiwan

## Abstract

*Introduction.* The lumbar puncture is a well-known neurological procedure. The purpose of this study is to build an accurate mathematical formula to estimate the appropriate depth for inserting a lumbar puncture needle for a beginner. *Methods.* This is a retrospective study of patients who underwent magnetic resonance imaging (MRI) of the L-spine. The depth from the skin to the posterior and anterior margin of the spinal canal at the level of L4-L5 and L3-L4 interspaces of the spine was estimated using MRI. *Results.* Three hundred sixty-eight patients aged between 20 and 89 years were studied. The optimal puncture depths of the lumbar puncture needle were moderately strongly related to weight and BMI. The most accurate models with the highest coefficient of determination were 1.27 + 0.18 × BMI and 1.68 + 0.067 × weight (kg) for man and woman, respectively. *Conclusion.* The best formula for men and women provides the most accurate estimates for adults based on the MRI of the L-spine.

## 1. Introduction

Lumbar puncture is an established but nevertheless difficult neurological procedure. It is a procedure to collect a sample of cerebrospinal fluid (CSF) for cytological, biochemical, or microbiological analysis. The majority of the procedures are for diagnosing meningitis and subarachnoid hemorrhage (SAH) [1]. The 10% to 20% incidence of traumatic lumbar puncture (needle-induced bleeding in the subarachnoid space) was shown in several studies [2, 3]. One relevant issue of lumbar puncture is how to successfully perform the procedure while maintaining the comfort of the patients. A lumbar puncture is typically performed at either the L3-L4 or L4-L5 interspace with a standard Quincke needle [4]. The appropriate depth for inserting the needle is a major problem. In 1988, Bonadio et al. determined that the depth of a lumbar puncture in children is positively related to the body surface area (BSA) [5]. When BSA increases by one unit, the depth of the lumbar puncture must increase by 2.56 cm. Six years later, a study of 586 children was conducted by Hasan et al. [6]. The depth was correlated with age and weight in older infants and children. In 1997, Craig et al. measured the depth of insertion of the lumbar punctures and recorded the demographic information of a group of children aged 0.01 to 16 years [7]. They found that the depth of insertion is positively related to the height of the patients. In 2005 and 2010, three studies determined that weight is vital for predicting the depth of lumbar punctures [8–10]. Most studies have examined the depth of lumbar punctures in children, and their data has included a range of ages [5–7, 10].

One-dimensional magnetic resonance imaging (MRI) was developed in the 1950s. Later, the 2D and 3D images were developed by Paul Lauterbur. Typically, MRI is used to differentiate between normal and pathological tissue. Unlike computer tomography (CT) scans and traditional X-rays, MRI does not involve harmful ionizing radiation. In 2005, Abe et al. studied the depth of lumbar punctures by using abdominal CT scans [9]. A model including the weight/height ratio was determined to be superior to four other reference models. Five regression models provided from 1994 to 2009 are shown in Table 1. Most of the models involved children, and one study involved patients aged from 25 days to 80 years. The smallest sample size was 54 and the largest sample size was 279.

In this paper, a more precise, MRI-based model was proposed and the models including different components were compared.

## 2. Methods and Materials

In this observational study, data from 590 patients who underwent L-spine MRI at Shuang Ho Hospital, Taipei Medical University, from January 2010 to August 2010 were recorded and measured. Sixty-five subjects who underwent lumbar laminectomy prior to MRI were excluded. In addition, 204 incomplete pieces of data were excluded. The final data pool consisted of 368 patients.

**Figure 1:**
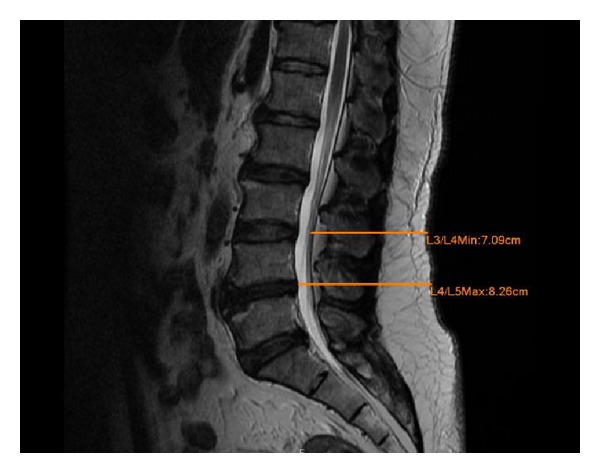
MRI of L-spine. Upper line is the posterior margin of the spinal canal to the skin (Min). Bottom line is the anterior margin of the spinal canal to the skin (Max).

For each patient, two distances (depth) to the skin were measured from his/her L-spine MRI. One is from the posterior margin of the spinal canal (minimal) and the other is from the anterior margin of the spinal canal (maximal). Lumbar puncture is typically performed in the L4 and L5 interspace while the patient is in the lateral position. Another option is performed in the L3 and L4 interspace, while the L4-L5 level cannot be easily performed. Here, we measured two distances in two interspaces. An example of measuring the depth is shown in Figure 1. The upper line and lower line represent the minimal depth of the L3-L4 level and the maximal depth of the L4-L5 level, respectively. In addition, the patient's age, height, and weight were collected from their medical records.

### 2.1. Statistical Method

First, the description of the data was present. The age, height, and weight between man and women were compared using Student's *t*-test. The association between the length of the needles and the factors was assessed by using Pearson correlation coefficients. The linear regression was used to predict the cerebrospinal fluid location. The correct rate of the lumbar puncture in this study is defined as a percentage of the predicted cerebrospinal fluid locations between their corresponding minimal and maximal depths. For all data, four models were compared. First and second models included weight and BMI. The last two models included weight/height and weight/height with gender, respectively. For each gender subgroup, three models were compared. They were weight, BMI, and weight/height. The correct rate of the lumbar puncture and the coefficient of determination (*R* ^2^) were determined to assess the accuracies of the models we compared.

### 2.2. Results

A retrospective cohort study from January 2010 to August 2010 was conducted with 590 patients who received MRI scans. Among them, 368 patients provided all the required information (e.g., height and weight). The range of age was 20 to 89 years and the average age was 58 years. There was no significant age difference between men and women (Table 2). There was no significant difference in both the maximal and minimal depths between men and women. A significant difference in height and weight was observed between men and women. The average height and weight of men were higher than that of women.

The associations between the target factors and the depths are shown in Table 3. The factor, weight, was moderately strongly related to the distance from the skin to the posterior and anterior margin of spinal canal. On the other hand, the factor, height, was significantly related to the distance from the skin to the posterior and anterior margin of the spinal canal. Another factor, age, was not related to the distance from the skin to the posterior margins of the spinal canal.

Four and three predicting models were compared for all data and for gender subgroups, respectively. The *R* ^2^ and correct rates of the predictions are shown in Table 4. The weight was a worst predictor according to its corresponding *R* ^2^ and success rate. For both L4-L5 and L3-L4, the regression model including the ratio and gender performed well according to their model *R* ^2^ and within rate. However, these factors predicted the depth of L3-L4 more accurately than that of L4-L5. When men and women were analyzed separately, the different predictor was found. At level L4-L5, the best predictor for men and women was the ratio of weight and height. At level L3-L4, the best predictors for men and women were BMI and weight, respectively.

**Figure 2:**
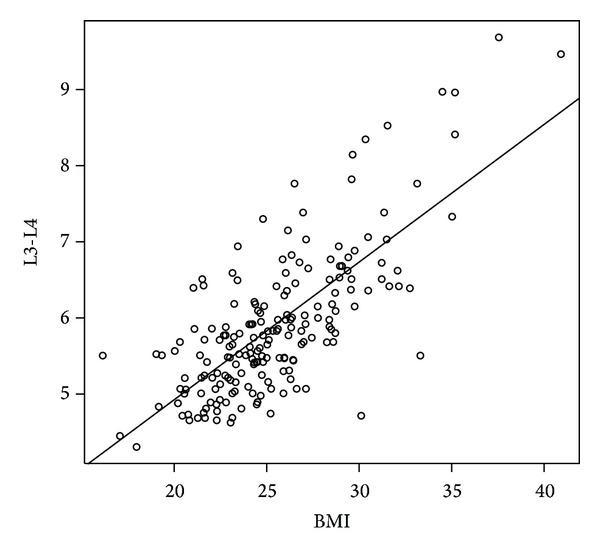
The scatter plot of BMI and median depth at level L3-L4 for men.

**Figure 3:**
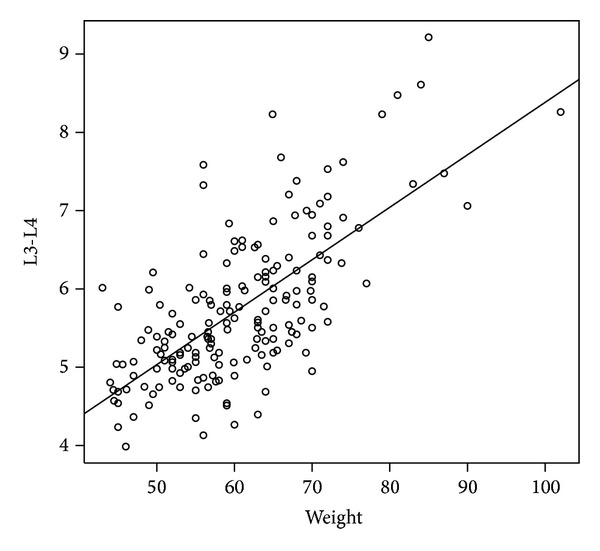
The scatter plot of weight and median depth at level L3-L4 for women.

The results of the predicted model for level L3-L4 are shown in Table 5 and the fitted lines were shown in Figures 2 and 3. For all participants, we found that the ratio of weight and height increased one unit and the depth at L3-L4 increased 0.1 cm. For men, when the BMI increased one unit, the depth at L3-L4 increased 0.182 cm. For women, when the weight increased one unit, the depth L3-L4 increased 0.067 cm.

## 3. Discussion

After a diagnostic lumbar puncture, the most common complication is headaches, especially for thin young women [11, 12]. To optimize patient care, the accurate selection of lumbar puncture needles is crucial. The selection of lumbar puncture needle may be more difficult for less experienced practitioners. The depth of lumbar punctures for children has been discussed in most studies on the topic. From several studies, the depth in children was found to be related to the body surface area [5–7], age, weight, and height (as shown in Table 1). Most of the models involved children, and one study involved patients aged from 25 days to 80 years. The smallest and largest sample sizes were 54 and 279, respectively. In our study, we recruited patients aged 20 years and older.

We observed that age was not significantly related to the distance from the posterior margin of the spinal canal at either the L3-L4 level or L4-L5 level. Height and weight were significantly correlated with the depths. The different predictors for men and women were found. The predictor BMI had the correct rate at 63.54% to estimate the depth for men. On the hand, weight was the best predictor for women with correct rate at 58.52%.

This study has 2 limitations. First, weight and height are typically easy to obtain, but not in an emergency department. Second, a measurement bias may have occurred. More than two times measurements could reduce the measurement bias. In future, other factors would be considered, such as wrist. Also, more complex model should be considered, such as polynomial regression.

In conclusion, the distance from the skin to the posterior or anterior margins of the spinal canal is more highly correlated with the weight/height ratio than age or height. The best formula to predict the available LP length at level L3-L4 for male is 1.27 + 0.18 × BMI and for female is 1.68 + 0.067 × weight (kg), and they were more reliable models for Taiwan males and females.

## Figures and Tables

**Table 1:** Demographic information of the 5 proposed models.

Author	Year	Age	Sample size	Model
Hasan	1994	Children (age not specified)	86	0.8 + 0.05 ∗ weight (kg)
Craig	1997	0 years–16 years	107	0.03 ∗ height (cm)
Stocker	2005	Children	54	1.8 + 0.05 ∗ weight (kg)
Abe	2005	25 days–80 years	175	1 + 17 ∗ weight/height (kg/cm)
Chong	2010	6 months–15 years	279	0.93 + 10.6 ∗ weight/height (kg/cm)

**Table 2:** Demographic information of data.

	*n* = 368	Men *n* = 192	Women *n* = 176	Mean difference
Age	58.00 (14.69)	57.37 (14.58)	58.69 (14.82)	−1.32
Height (cm)	161.80 (8.44)	167.06 (6.58)	156.04 (6.21)	11.01∗
Weight (kg)	66.20 (12.72)	71.32 (12.93)	60.58 (9.79)	10.74∗
L4-L5				
Minimal depths	5.54 (1.04)	5.54 (1.03)	5.53 (1.06)	0.01
Median	6.07 (1.03)	6.08 (1.01)	6.06 (1.04)	0.03
Maximal depths	6.60 (1.03)	6.62 (1.02)	6.58 (1.05)	0.04
L3-L4				
Minimal depths	5.30 (0.98)	5.38 (0.97)	5.22 (0.98)	0.16
Median	5.83 (0.95)	5.90 (0.94)	5.74 (0.95)	0.16
Maximal depths	6.35 (0.94)	6.42 (0.93)	6.26 (0.94)	0.16

Values in table: mean (standard deviation); *P value less than 0.05.

**Table 3:** Pearson correlation coefficients between factors and depths.

	Posterior margins	Median	Anterior margins
L4-L5			
Age	−0.087	−0.108∗	−0.127∗
Height	0.115	0.136∗	0.154∗
Weight	0.596∗	0.610∗	0.609∗
BMI	0.653	0.656	0.643
*W*/*H* ^+^	0.650∗	0.659∗	0.653∗
L3-L4			
Age	−0.100	−0.115∗	−0.129∗
Height	0.179∗	0.182∗	0.181∗
Weight	0.663∗	0.667∗	0.658∗
BMI	0.692	0.695	0.684
*W*/*H* ^+^	0.707∗	0.711∗	0.700∗
*Men *			
L4-L5			
Weight	0.675∗	0.697∗	0.703∗
BMI	0.689∗	0.703∗	0.713∗
*W*/*H* ^+^	0.685∗	0.691∗	0.705∗
L3-L4			
Weight	0.710∗	0.732∗	0.738∗
BMI	0.719∗	0.737∗	0.746∗
*W*/*H* ^+^	0.713∗	0.724∗	0.737∗
*Women *			
L4-L5			
Weight	0.648∗	0.609∗	0.650∗
BMI	0.657∗	0.608∗	0.654∗
*W*/*H* ^+^	0.654∗	0.595∗	0.646∗
L3-L4			
Weight	0.694∗	0.646∗	0.693∗
BMI	0.690∗	0.648∗	0.691∗
*W*/*H* ^+^	0.672∗	0.637∗	0.677∗

^+^Ratio of weight to height; *P value < 0.05.

**Table 4:** The coefficient of determination (*R* ^2^) of different models and their corresponding success rate.

	Predictor	*R* ^2^	Success rate, %
L4-L5	Weight	0.37	49.2
	BMI	0.43	50.0
	Weight/height	0.43	51.6
	Weight/height + gender	0.46	54.4
L3-L4	Weight	0.44	56.5
	BMI	0.48	56.8
	Weight/height	0.51	59.2
	Weight/height + gender	0.52	59.2

*Men *			
L4-L5	Weight	0.47	52.1
	BMI	0.49	54.2
	Weight/height	0.51	55.2
L3-L4	Weight	0.52	58.3
	BMI	0.54	63.5
	Weight/height	0.55	60.4

*Women *			
L4-L5	Weight	0.43	47.7
	BMI	0.37	44.9
	Weight/height	0.42	47.2
L3-L4	Weight	0.47	58.5
	BMI	0.42	49.4
	Weight/height	0.47	56.3

Success rate: percent of the predicted cerebrospinal fluid location between the minimal and maximal depths.

**Table 5:** Estimators for the depth at L3-L4.

Variables	Estimate	SD	*t* value	*P* value
Intercept	1.731	0.214	8.083	<0.001
Weight/height	0.100	0.005	19.364	<0.001

Men				
Intercept	1.272	0.311	4.083	<0.001
BMI	0.182	0.012	15.018	<0.001

Women				
Intercept	1.682	0.327	5.142	<0.001
Weight	0.067	0.005	12.567	<0.001
